# Religion and perceptions of community-based conservation in Ghana, West Africa

**DOI:** 10.1371/journal.pone.0195498

**Published:** 2018-04-05

**Authors:** Grant Murray, Andrew Agyare

**Affiliations:** 1 Division of Marine Science and Conservation, Nicholas School of the Environment, Duke University Marine Lab, Beaufort, North Carolina, United States of America; 2 Ghana Wildlife Division of the Forestry Commission, Accra, Ghana; Emory University, School of Public Health, UNITED STATES

## Abstract

Adapting community-based protected areas to local context and evaluating their success across a range of possible socio-economic and ecological outcomes depends, in part, on understanding the perceptions of local actors. This article presents results from a mixed methods study that focuses on how and why religious identity, a prominent aspect of Ghanaian culture, is related to perceptions of the performance of several Community Resource Management Areas (CREMAs). CREMAs are a form of Ghanaian protected area that emphasizes community participation and a range of socio-economic and ecological objectives. Using importance-satisfaction analysis, large-scale survey results show that respondents that identify as Christians consistently assign greater importance to CREMA outcomes than do those that identify with Traditional religions. Education and whether respondents were native to an area (both of which were correlated with religious identity) were also systematically related to perceptions of CREMA importance, with those that are educated and non-native to an area tending to assign higher importance to CREMA outcomes. Follow up focus group participants from the Avu Lagoon CREMA suggest that the patterns result from differing ‘openness’ to new ideas, relative dependence on natural resources, acceptance of Traditional practices associated with conservation, and a sense, for some, that ecological conditions are divinely ordained. Christianity, education and non-nativity are associated with much larger performance gaps, particularly with respect to socio-economic impacts. The article concludes with a discussion of the implications for conservation interventions and the use of perceptions in assessing protected area performance.

## Introduction

Recent decades have seen a growth in protected area (PA) types that attempt to meaningfully engage local communities in governance regimes and/or to deliver a range of socio-economic outcomes in addition to core biodiversity goals [1–2]. Adapting community-based PAs to local context, as well as evaluating their success, however, remains both a priority and a challenge, given the multiple socio-economic, organizational and ecological objectives and outcomes that are often associated with community-based PAs [3–7]. To address this challenge, researchers sometimes focus on the perceptions of local community members for a range of reasons, including adapting interventions to locally salient priorities/objectives, assessing community level support for and the perceived legitimacy of conservation interventions, understanding socio-cultural and economic impacts and how they are distributed, and obtaining a detailed view of local-level ecological impacts [8]. This article contributes to work on local level perceptions by focusing on the relationship between religion–a key aspect of life in Ghana and other parts of sub-Saharan Africa—and perceptions of the performance of several Community Resource Management Areas (CREMAs) across Ghana.

Like other countries in sub-Saharan Africa, Ghana has increasingly turned to decentralized and/or community-based forms of protected areas as a means of addressing linked social and ecological goals [9]. The CREMA program, established in 2000, represents an attempt to strengthen relationships between traditional and state authorities, as well as to increase local level participation in the management of natural resource [9–12]. CREMAs are designed using flexible guidelines and often involve several constituent communities that group together to manage shared or adjacent lands [10–11]. At the time of this writing, Ghana had 32 CREMAs distributed throughout the country, 24 of which have received their final certificate of devolution. Using Importance-Satisfaction analysis to focus on community perceptions of CREMA performance, previous research has shown wide variability at both the CREMA and constituent community (single community) levels [10–11]. This research, however, does not empirically address the possible explanatory factors for that variability.

Other relevant work related to conservation and PAs in Ghana (as well as other parts of sub-Saharan Africa) suggests that Traditional religious beliefs and practices are relevant to conservation interventions [13–19]. ‘Traditional religion’ is a broad term that encompasses a range of religio-spiritual beliefs, institutions and actors such as priests/priestesses, taboos, and gods/divinities that are often related to the land and waters [16]. Generally, taboos are described as unwritten rules that regulate behaviour. They can often be associated with the non-human (spiritual) world and can regulate such things as forest clearing and the hunting and/or eating of particular species. Taboos are sometimes associated with particular objects (sometimes called gods or divinities) such as individual species, places, or water bodies, and have priests/priestesses associated with those objects [16]. Ntiamoa-Baidu (1991), for example, describes taboos associated with coastal lagoons including, *inter alia*, gear restrictions, weekly temporal restrictions (e.g. no fishing on Friday mornings and Tuesdays), and closed seasons [20].

The connections between religion and conservation in Ghana are clearly not limited to Traditional religions, nor are Traditional religions the dominant Ghanaian religious group. In addition to the approximately 5% of Ghanaians that self identify as believers in Traditional religions, 71.2% of identify as Christian, and 17.6% as Muslim [21]. Religions of all types are an important aspect of Ghanaian culture. For example, The Economist (2012), citing a Win-Gallup poll, recently called Ghana the most devout country in the world, with 96% of the population professing to be religious [22]. In at least one case, NGOs have made a clear link between Christianity and CREMAs (and, more generally, the imperative to conserve). A Rocha Ghana (ARG), for example, is an NGO with a Christian background that has worked with teams of Christian and Islamic scholars to help develop the Murugu-Mognori CREMA, adjacent to Ghana’s flagship Mole National Park [23].

Whether the focus is on Traditional religions or Christianity, however, the emphasis of much work at the religion-conservation nexus in Africa/Ghana is on identifying the specific religio-cultural institutions and beliefs that can serve as entry points or can be leveraged for conservation interventions [17, 23–24]. Less attention has been paid to how religion/religious beliefs, as a core element of Ghanaian culture, might shape perceptions of, receptivity towards, and/or evaluations of conservation interventions, including CREMAs. Moreover, there has been relatively little national scale studies centered on perceptions of community-based conservation interventions in Ghana, or elsewhere for that matter [8]. In this context, the goals of this study were 1) to systematically characterize the role of religious identity in shaping perceptions of CREMA performance in Ghana, and 2) to explain why/how religion is shaping those perceptions.

## Materials and methods

The Ethics Review Board at Vancouver Island University (Canada) approved this study. Approval number 885-2008-1003.

### Background and site description

As described below, the Avu Lagoon CREMA was the site of follow up qualitative work designed to help interpret earlier survey results. As such, some additional details about this study site are provided here. Due to an abundance of swamps and regular flooding, the Avu Lagoon area is relatively sparsely populated and most of the constituent communities are remote from urban areas. The Avu Lagoon CREMA encompasses 15 communities including: Agorbledokui, Avuto, Akutukope, Bekpo, Blemeazado, Bludo, Tsawoeme, Wenu, Adutor, Agbagorme, Bayive, Gui, Suipe, Tosukpo, Xavi, and Agbogbla [25]. The land around the Avu Lagoon is owned by several clans and is subdivided into individual family holdings. Each clan has its own chief with rules and community gods to worship, with some villages having multiple clans [25]. Major activities include farming, fishing, hunting, collection of firewood, weaving and the distillation of akpateshie (a distilled liquor produced from sugar cane). The Avu Lagoon CREMA was created in 2006 through the collaborative efforts of the Wildlife Division and the Nature Conservation Research Center; major goals of the NCRC included the protection of the rare sitatunga (*Tragelaphus spekii gratus*) and the development of alternative livelihoods, including ecotourism [19]. Initial efforts included training selected local community members as an Environmental Education Team to raise awareness about the potential of Avu Lagoon for conservation and ecotourism. Christian church platforms were sometimes used for these efforts.

### Data collection

**Fig 1:**
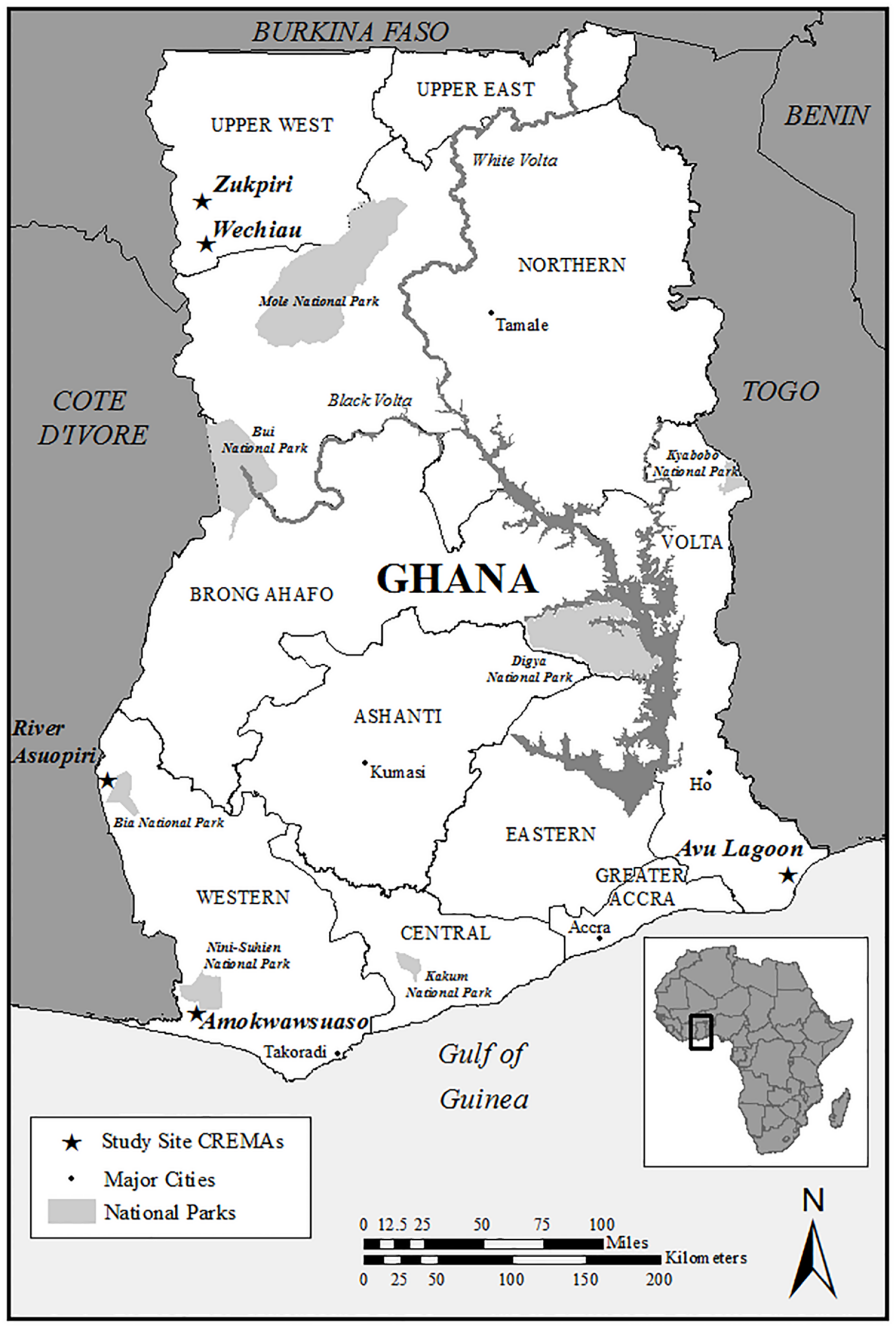
Location of the five CREMA sites in Ghana.

This research was conducted at two, complementary scales (national and local), involving a mix of qualitative and quantitative elements. Five CREMAs were selected through a stratified (by major land tenure systems) random sampling procedure (Fig 1). Selected CREMAs included Avu Lagoon, Zukpiri, River Asuopiri, Amokwawsuaso and Wechiau. Each of these CREMAs is associated with multiple constituent communities. The sites are geographically dispersed across the country, and feature a range of ethnicities, distances from urban centers, ecosystem types, socio-cultural composition of the constituent communities, tenure regimes/traditional chieftainship, and religious makeup [10–11].

### Initial qualitative work

Initial interviews and focus groups were largely focused on informing survey design and specifically to develop a list of possible desired outcomes from CREMA implementation. The initial qualitative phase involved 60 interviews (17 non-local senior personnel in policy and practise, and 43 involved with the five CREMAs) and 20 focus groups (4 in each community). The first key informants were selected through expert advice from knowledgeable researchers with the rest selected through referrals. Focus groups were composed of six to eight persons each, representing key aspects of local economies and social groups: enterprise development groups; farmers; fishermen, hunters, youth, Non Timber Forest Products (NTFP) gatherers, and women. Among other things, this initial qualitative phase focused on identifying the outcomes stakeholders hoped that CREMAs would produce.

### Surveys

The survey was administered over a five-month period (April—August 2012) to 929 respondents in 37 constituent communities in the 5 selected CREMAs. This included 232 respondents at the Avu Lagoon CREMA; the results of this portion of the survey are the focus of this article, though detailed larger scale survey results are provided online as supplemental information. Efforts were made to randomize this process, though some accommodation had to be made based on logistics in remote communities. Individuals in selected households were identified to take the survey, with men and women alternately selected. At the national level a total of 17 research assistants (most of whom were teachers) were used for data collection. Survey questions regarding CREMA perceptions were designed using an importance-satisfaction analysis design [10–11, 26–27]. Building on the initial qualitative phase, the survey contained a list of 28 items that were mentioned as possibly desired outcomes from the establishment of CREMAs. For each of the 28 outcomes, individuals were first asked to rate, using a Likert-style scale from 1–5, how important each outcome was (with 5 being most important). A follow-up question then went back through each outcome and asked individuals to again rate from 1–5 how satisfied they were with the achievement of each outcome (with 5 being most satisfied). In importance satisfaction analysis, attention is drawn to the ‘gap’, or difference between importance and satisfaction ratings for the same item. Items with larger ‘gaps’ are associated with areas more in need of management attention [26–27].

Survey data were analyzed using SPSS, and differences between mean scores for different groups (e.g. Christians vs. Traditionalists and those with schooling vs. those without) on the scaled questions were tested for significance (at the p = .05 level) using Student’s t-test. Mean scores were calculated for both importance and satisfaction measures, and the differences between means were calculated to derive performance gaps.

### Subsequent focus groups

In order to help interpret some survey results related to religion and education, a series of focus groups were later (2015) held at the Avu Lagoon CREMA site. Avu Lagoon was selected for follow-up focus groups because it had a relatively high proportion of respondents self-identifying as following a ‘Traditional’ religion. It also did not have many respondents who identified as Muslim (only 1), and most respondents said that they were native to the area (see Table 1). Together, this served to simplify discussions and allow for a focus on differences between only two religions along with the role of education. Two focus groups were held in each of four constituent communities: Adutor, Avuto/Akutukope, Agorbledokui, and Wenu. These particular communities were selected based on a combination of logistics as well as religious composition and levels of education, as indicated in the initial survey findings (Table 1). For example, Wenu was chosen because it is largely Traditionalist (100% of survey respondents), Adutor was chosen because it is largely Christian (88% of survey respondents), while Agorbledokui and Avuto/Akutukope were a more even blend of both religions. As suggested by Table 1, these communities also differed in terms of levels of schooling and ‘nativity’.

**Table 1:** Demographic profile of survey respondents.

Characteristic	Overall (all CREMAs)	Overall (Avu)	Avu Focus Group Communities			
			Adutor	Agor-bledokui	Avuto/ Akutukope	Wenu
**No School**	424 (45.9%)	71 (30.6%)	8 (18.1%)	4 (26.7%)	8 (40%)	7 (63.6%)
**Some school**	500 (54.1%)	161 (69.4%)	36 (81.8%)	11 (73.3%)	12 (60%)	4 (36.4%)
**Missing—school**	5	0	0	0	0	0
**Christian**	415 (81.4%)	135 (77.1%)	39 (88.6%)	7 (50%)	12 (75%)	0 (0%)
**Traditionalist**	61 (12%)	39 (22.3%)	3 (6.8%)	7 (50%)	4 (25%)	11 (100%)
**Muslim**	34 (6.7%)	1 (.6%)	1 (2.3%)	0	0	0
**Missing—religion**	419*	57*	1	1	4	0
**Native**	606 (86.7%)	228 (98.3%)	44 (100%)	15 (100%)	20 (100%)	11 (100%)
**Non-native**	93 (13.3%)	4 (1.7%)	0	0	0	0
**Missing—nativity**	230*	0	0	0	0	0

* high item non-response was due to the length of the survey. Item non-response did not systematically skew the data.

In all cases, focus groups were comprised of an approximately equal balance of adult males and females. In all but two cases, there were 8 participants in each group (there were 6 in one group, and 7 in a second). All focus groups were balanced to include an approximately equal number of Christians and Traditionalists (except the Wenu groups), and of educated and non-educated individuals in each of the two religious categories. Given the survey results showing very few non-natives in the Avu Lagoon constituent communities, no attempt was made to balance composition between those that considered themselves native to the area and those that did not. Focus groups began with a brief overview of key patterns from the survey results in general terms (see below) followed by a series of questions asking participants to reflect on and explain those patterns. Focus groups were held in the local language and were conducted and translated into English with the help of three trained assistants.

### Limitations

In considering the findings below, it is important to note some limitations of the study. First, the study focuses on a limited set of characteristics (nativity, education, and religion) but other factors almost certainly play a role in shaping perceptions of CREMA performance [10–11]. It is important to not oversimplify, or to assign direct or simple causality between these characteristics and perceptions of CREMA performance. Second, our sampling strategies and item non-response challenges (see supplemental information and Table 1 above) resulted in the potential for biased results and an inability to fully consider Ghana’s third major religion: Islam. On the other hand, while item non-response rates for religion and nativity were high, these rates resulted from the question not being asked, rather than respondent refusal (suggesting systematic bias was not injected). Moreover, survey patterns were strongly supported by focus group results, as indicated below. Third, giving respondents the close-ended choice of self-identifying as either Christian or Traditional may present a false binary, given the ‘hybrid’ religious practices of some individuals [23, 28]. Fourth, while the focus groups suggest a role between Traditional beliefs/practices and CREMA perceptions in the Avu Lagoon case study, we do not have full information about what specific Traditional rules/norms might be associated with lands/objects that are covered by the Avu Lagoon (and other CREMAs), and what level of congruence there is between those guidelines and CREMA rules (*cf* [19]). Relatedly, we do not have information as to whether these Traditional beliefs/practices have conservation as a primary goal, or whether conservation effects are ancillary to other cultural goals and social needs such as safety, health, or ancestor worship [29–30]. Finally, our analysis here is focused on patterns of response across all 28 outcomes based on demographic characteristics, rather than analyzing variability or patterns in the relative importance or satisfaction assigned to individual outcomes themselves (however, see [10–11] for analysis of this sort). Each of these limitations suggests important areas for follow up research.

## Results

### Surveys

Table 2 displays mean importance and satisfaction scores for two categories of respondents at Avu Lagoon (those that identified as Christian, and those that identified as Traditionalist) for each of the 28 outcomes, and shows the performance gap for each category of respondent. Outcomes are arranged by descending magnitude of the performance gaps for Christians. Importance scores for Christians were higher in all but one case (except ‘more bushmeat’), and significantly so in 24 of 28 cases. Satisfaction scores for Christians were higher in all but 3 cases, and significantly so in 20 of 28 cases. In addition to these consistent differences between Christians and Traditionalists, there are other major patterns visible in the results, which are analyzed in more detail elsewhere [10–11]. For example, the four outcomes for which there were not statistically significant differences in importance scores were ‘improved supply and quality of firewood and charcoal’, ‘more fish’, ‘more bushmeat’, and ‘increased conservation awareness’. The first three were a result of lower (as compared to other items) importance scores for Christians, while the last one was a result of higher scores for Traditionalists. Interestingly, these areas were major areas of emphasis for the Nature Conservation Research Center, the Ghanaian NGO that was active in the area and in the establishment of the CREMA [10–11, 19]. The performance gaps for Christians (mean = 1.28) were, on average, approximately twice as large as for Traditionalists (mean = .68). It is also worth noting that, while there were performance gaps for all outcomes, the largest gaps were strongly associated with socio-economic outcomes (those at the top of Table 2).

**Table 2:** Mean importance /satisfaction scores and performance gaps for Christians (C) and Traditionalists (T) at Avu Lagoon CREMA (n = 174)^a^.

Outcomes^b^	Importance			Satisfaction			Gaps	
	C^c^	T^d^	p^e^	C	T	p^e^	C	T
**educational scholarships**	4.4	2.82	<**0.001**	2.5	1.97	**.013**	1.9	0.85
**increased income**	4.32	2.82	<**0.001**	2.66	2.03	**.003**	1.66	0.79
**capacity building/training in income generating enterprises**	4.19	3.21	<**0.001**	2.78	2.59	0.446	1.41	0.62
**access to credit/financial assistance**	4.15	2.62	<**0.001**	2.76	1.97	**.001**	1.39	0.65
**increased employment**	4.31	2.92	<**0.001**	2.93	2.28	**.004**	1.38	0.64
**improved social infrastructure**	4.21	2.95	<**0.001**	2.85	2.15	**.001**	1.36	0.8
**constancy of kids school attendance**	4.11	2.85	<**0.001**	2.81	2.13	**.003**	1.3	0.72
**more poles and construction materials**	4.11	3.36	<**0.001**	3.13	2.67	**.020**	0.98	0.69
**more and better quality traditional medicines**	4.14	3.18	<**0.001**	3.2	2.72	**.012**	0.94	0.46
**improved supply and quality of firewood and charcoal**	3.31	3.18	.507	2.44	2.56	0.548	0.87	0.62
**improved water supply and quality**	4.26	3.64	**0.001**	3.39	3.05	0.131	0.87	0.59
**more and better quality grass**	4.18	3.64	**0.004**	3.35	3.13	.230	0.83	0.51
**tourism**	4.53	3.38	<**0.001**	3.82	3.15	**0.001**	0.71	0.23
**fodder for livestock**	3.91	3.05	<**0.001**	3.21	2.46	**0.001**	0.7	0.59
**more fish**	3.76	3.44	0.152	3.08	3	0.691	0.68	0.44
**religious, cultural and historical uses**	4.13	3.59	**0.017**	3.45	3.26	0.385	0.68	0.33
**better farmlands, increased food production**	4.34	3.61	<**0.001**	3.68	3.13	**0.01**	0.66	0.48
**international recognition and pride**	4.35	3.56	<**0.001**	3.76	3.33	**0.047**	0.59	0.23
**ecologically sensitive areas protected and well managed**	4.41	3.74	**0.001**	3.83	3.67	0.485	0.58	0.07
**wind break**	3.6	2.82	**0.001**	3.03	2.64	**0.041**	0.57	0.18
**native wildlife return**	4.36	3.64	**0.001**	3.79	3.38	**0.049**	0.57	0.26
**no chemical contamination of water**	4.08	2.9	<**0.001**	3.52	2.67	<**0.001**	0.56	0.23
**increased conservation awareness**	4.43	4.28	0.428	3.87	3.92	0.759	0.56	0.36
**purification and provision of clean air**	4.26	3.33	<**0.001**	3.72	2.9	<**0.001**	0.54	0.43
**collective community action and unity**	4.08	3.13	<**0.001**	3.55	2.95	**0.004**	0.53	0.18
**more rain**	3.89	3.26	**0.003**	3.39	2.82	**0.005**	0.5	0.44
**reduced bush fires**	4.39	3.41	<**0.001**	3.98	3.13	<**0.001**	0.41	0.28
**more bushmeat**	2.46	2.62	0.546	2.07	2.1	0.885	0.39	0.52
**Average performance gap (all outcomes)**							1.28	0.68

^a^ In 57 cases there was no response recorded for this question. This was due to the length of the survey and item non-response did not systematically skew the data.

^b^ Outcomes arranged by decreasing magnitude of performance gaps for Christians.

^c^Respondents that self-identify as Christian (n = 135).

^d^Respondents that self-identify as Traditional (n = 39).

^e^ items in bold are significant at the p<.05 level.

Table 3 displays mean importance and satisfaction scores for two categories of respondents (those that identified as having some school education, and those that identified as not having any school education) for each of the 28 outcomes, and shows the performance gaps for each category of respondent. Outcomes are arranged by descending order of magnitude of the performance gaps for those that identified as having some school education. The mean importance scores for those that identified as having some school education were larger in all 28 cases, and significantly so in 16 of 28 cases. Mean satisfaction scores were higher for 25 of 28 outcomes for those that identified as having some school education, but these differences were relatively small, and only significant in one case. Performance gaps were larger for those with some school education, but the differences were relatively small. Again, the performance gaps for socio-economic outcomes tended to be largest.

**Table 3:** Mean importance /satisfaction scores and performance gaps for those with school (S) and no school (NS) at Avu Lagoon CREMA (n = 232).

Outcomes^a^	Importance			Satisfaction			Gaps	
	S^b^	NS^c^	P^d^	S	NS	p	S	NS
**educational scholarships**	4.16	3.65	**.022**	2.61	2.56	0.843	1.55	1.09
**increased income**	4.07	3.63	**.045**	2.69	2.68	0.944	1.38	0.95
**improved social infrastructure**	4.04	3.54	**.020**	2.83	2.76	0.755	1.21	0.78
**increased employment**	4.10	3.69	.067	2.94	2.87	0.736	1.16	0.82
**capacity building/training in income generating enterprises**	3.97	3.80	0.376	2.82	2.96	0.494	1.15	0.84
**access to credit/financial assistance**	3.93	3.39	**.017**	2.8	2.55	0.235	1.13	0.84
**constancy of kids school attendance**	3.84	3.44	**0.060**	2.83	2.5	0.1	1.01	0.94
**more poles and construction materials**	4.03	3.65	**0.022**	3.19	3.15	0.859	0.84	0.50
**more and better quality traditional medicines**	3.98	3.79	0.297	3.21	3.03	0.32	0.77	0.76
**improved water supply and quality**	4.14	3.77	**0.018**	3.4	3.24	0.357	0.74	0.53
**improved supply and quality of firewood and charcoal**	3.24	3.13	0.526	2.51	2.54	0.849	0.73	0.59
**more and better quality grass**	4.11	3.79	**0.039**	3.46	3.31	0.385	0.65	0.48
**fodder for livestock**	3.81	3.42	**0.015**	3.17	3.04	0.463	0.64	0.38
**tourism**	4.34	3.96	.053	3.77	3.67	0.581	0.57	0.29
**more fish**	3.65	3.39	0.155	3.09	3.08	0.958	0.56	0.31
**better farmlands, increased food production**	4.22	3.87	**0.022**	3.67	3.37	0.075	0.55	0.50
**international recognition and pride**	4.25	3.82	**.031**	3.73	3.49	0.186	0.52	0.33
**religious, cultural and historical uses**	3.99	3.76	0.209	3.49	3.37	0.492	0.50	0.39
**increased conservation awareness**	4.40	4.25	0.315	3.91	3.94	0.832	0.49	0.31
**purification and provision of clean air**	4.14	3.70	**0.005**	3.69	3.39	0.066	0.45	0.31
**no chemical contamination of water**	3.87	3.54	**0.074**	3.43	3.25	0.298	0.44	0.29
**collective community action and unity**	3.94	3.52	**.044**	3.5	3.27	0.195	0.44	0.25
**ecologically sensitive areas protected and well managed**	4.26	3.92	**0.038**	3.84	3.68	0.305	0.42	0.24
**reduced bush fires**	4.26	3.89	**0.014**	3.86	3.68	0.237	0.40	0.21
**native wildlife return**	4.22	3.79	**0.015**	3.84	3.54	0.08	0.38	0.25
**more rain**	3.79	3.41	**0.021**	3.44	3.23	0.176	0.35	0.18
**wind break**	3.42	3.04	0.055	3.09	2.72	0.015	0.33	0.32
**more bushmeat**	2.47	2.21	0.198	2.2	1.9	.044	0.27	0.31
**Average performance gap (all outcomes)**							0.70	0.50

^a^ Outcomes arranged by decreasing magnitude of performance gaps for those with some school (S).

^b^ Respondents that identify as having some schooling (n = 161).

^c^ Respondents that identify as having some schooling (n = 71).

^d^ items in bold are significant at the p<.05 level.

At the Avu Lagoon level, there was a correlation between religion and education (n = 174); Traditionalists were more likely to have had no schooling than Christians (Pearson’s chi-square = 23.221, df = 1; p<.001). This was a moderate to strong correlation (phi = -.365).

For space reasons, survey results at the national level (which involved 929 respondents across five CREMAs) are presented as supporting information. Briefly, response patterns at the national level strongly echoed those at the single CREMA (Avu Lagoon) level, with Christian (S1 Table) and educated (S2 Table) respondents assigning systematically higher importance scores and, to a somewhat lesser extent, satisfaction scores to the same list of 28 outcomes. In addition, the information in S3 Table shows that respondents that described themselves as non-native to the area assign systematically higher importance scores than respondents that described themselves as native to the area. S1 Text shows that, as at Avu Lagoon, these three characteristics are correlated.

### Focus groups on the role of religion and education in shaping CREMA attitudes

Before asking about religion or education specifically, focus group respondents were asked to reflect on why there were observed differences in survey response patterns among the various (15) constituent communities that participate in the Avu Lagoon CREMA. Some of the responses here had little to do with religion or education. For example, in Avuto/Akutukope #2 and Agorblodokui #1 and #2 there was general agreement that community perceptions of CREMA importance depended on how far the community is from the Lagoon, with those closer to the Lagoon seen as being more dependent on it, and therefore having a greater need to conserve it. Respondents in Wenu #1, on the other hand, pointed to the process of CREMA creation, and felt that differences among communities might have to do with the level of interaction with a group set up by Environmental Education Team (a group organized by the Nature Conservation Research Center). Others pointed to the fact that different communities had different ‘agendas’ and different livelihood strategies.

One of the main themes, however, that emerged had to do with the perceived level of ‘development’ of the community. This was sometimes framed in economic terms, but also in terms of how ‘open’ communities were and how much interaction they had with different types of people. In turn, some respondents explained that how ‘progressive’ or ‘open’ a community is was related to ‘diversity’, which was variously described in terms of education levels, or the amount of in-migration to the area (exposure to non-native peoples). Openness was also connected to religion, with respondents in Avuto/Akutukope #2, for example, expressing that a lack of religious diversity was related to how ‘conservative’ a community is. The information in appendix S1 Text shows that education and religion are correlated.

Religion was consistently seen as an important part of identity at the personal and community levels, and at a general level was associated with beliefs about appropriate ways to interact with Nature. Respondents also generally agreed that religion plays a role in shaping attitudes towards conservation in general and CREMAs in particular. Some of these comments were not necessarily specifically associated with either religion–for example, many respondents stated that religion fosters ‘good moral values’, or provides spiritual guidelines about how to appropriately interact with the non-human world. More than one theme emerged as to how specific religions shaped attitudes towards Nature generally, and with respect to conservation and CREMAs in particular. For example, Christianity was seen by some to be correlated with higher levels of education and a more modern and open worldview. This was provided as a reason for explaining why Christian survey respondents attached more importance to conservation outcomes. On the other hand, there was a common sentiment that Christians do not ‘toe the line’–that they tend to be firm in their beliefs and resistant to Traditional teachings, including those related to beliefs and practices associated with the non-human world. To illustrate this point, some respondents spoke about elders attempting to teach Christians about the importance of certain taboos, but that Christians were ‘adamant’ about not listening/believing because those beliefs were not congruent with Christian tenets. For some respondents, this was seen as part of a larger perception that Christians are less connected to the natural world, less directly dependent on natural resources, and more ‘liberal’ with resource usage, all of which were seen to cause environmental problems.

Traditionalists, on the other hand, were widely described as ‘closer’ to resources, more dependent on them and, in many cases, more knowledgeable about them. This connection with the land was associated by some with an ability to provide more accurate assessments about the condition of the land and the effects of CREMAs. A respondent in Agorblodukui #1, for example, noted that Christians have learned a lot about conservation in ‘churches and schools’, but

“…on the other hand, we [Traditionalists] have been conserving the environment ourselves for a long time; we see changes much better than Christians […] it is we the Traditionalists–hunters—who mostly lead people to where the animals can be located in the CREMA and get paid for our labor, but the Christians are not involved much in such work and therefore they cannot see the achievements of the CREMA better than us”.

Some respondents felt that Traditional religious beliefs were important in terms of managing resources, and noted the importance of particular taboos against destroying particular objects, such as ‘trees, plants, river bodies and other things.’ At the same time, some respondents communicated a sense that Traditional beliefs and practices are rigidly ‘handed down’ without questioning or understanding, and that strict adherence to religious practices left little room for efforts to raise awareness that might ‘interfere’ with Traditional ways of interacting with the natural world, thereby challenging the adoption of new ideas and new practices (including CREMAs). In several focus groups, respondents noted that Traditionalists ‘take (conservation) outcomes for granted.’ This was somewhat difficult to interpret, but seemed to be related to a sense that natural resources are provided through divine intervention and not necessarily through (outside) conservation interventions such as CREMAs. For example, a respondent in Wenu #1 (a largely Traditionalist community) stated that:

“because of the much more frequent encounter with natural resources by the uneducated they would probably take the CREMA outcomes as a given and hence do not attach as much importance to them [as the results of CREMAs] as the educated world would”

In some focus groups, respondents pointed to particular religious practices that provide a mechanism for furthering the conservation agenda. Respondents spoke, for example, about using ‘biblical messages’ to support conservation messaging and education. Others pointed to Traditional rituals (particularly sacrifices and libations) that are thought to lead to better natural resource conditions, and also that non-observance incites ancestral wrath with dire consequences not only for the individual but the family or clan as a whole. Others pointed out that Christians do not believe in these rituals and do not participate in ‘performances.’

Like religion, education was also consistently seen to play a role in shaping conservation perceptions, and in explaining survey response patterns. Some respondents in Adutor, for example, described being taught in school about the importance of trees and conservation in general, while explaining that those that who do not attend school are taught by their parents (who are seen as mostly practicing Traditionalists). Respondents in Avuto/Akutukope #2 noted that education creates a broader awareness, and openness to new ideas. Interestingly, some respondents noted that school education raised expectations (this was echoed in Wenu #1), and created a desire for ‘quick results’–this was seen to partially explain large performance gaps. Other respondents (also in Wenu #1) noted that school education provided opportunities (economic/livelihood) that freed individuals from direct dependence on natural resources, and might therefore make them more likely to support conservation measures that (are perceived to) curtail resource usage. Similarly, respondents noted that non-school-educated individuals tend to rely more directly on natural resources for their livelihood, and might therefore fear loss of access to resources. Others felt that school education also raised awareness of, and hope for, economic gain and the development of new opportunities including, in particular, eco-tourism and attendant economic prospects.

Although the survey at Avu Lagoon suggested a low percentage of non-natives in the area (see Table 1 above), focus group respondents were asked to comment on the fact that at the national level non-natives generally had similar patterns to Christians and those with some school education. Generally, non-natives were seen to be more ‘experienced’, and to have a higher awareness of conservation issues (and therefore to assign higher values to them). Some respondents also expressed a feeling that non-natives had a wider set of livelihood options, and were therefore able to make more conservation-oriented decisions.

## Discussion and conclusions

In this study religion, education and nativity were consistently associated with differing perceptions of CREMA performance (as measured by importance and satisfaction ratings). These patterns held at both the national (all 5 CREMAs) and Avu Lagoon levels. Analysis of the survey findings shows that these demographic characteristics are correlated in statistically significant ways—a relationship that was echoed in the focus group discussions. Indeed, at times the characteristics were used somewhat interchangeably as markers of two groups: 1) native, Traditional religion adherents who are not formally educated, and 2) more highly/formally educated Christians who are not native to the area. The concepts of ‘development’ and ‘openness’ were often used to denote differences between these two groups, with the former seen as less developed and/or open than the latter. The systematic trends in responses by Christians, non-natives and those that are educated suggests that there is more importance placed by this group on the CREMA mechanism as a way forward and, more generally, that there is a need to find ‘new’ ways to manage resources. Focus group results suggest that this is based on openness to new ideas, less direct dependence on natural resources, and a lack of faith in Traditional practices. On the other hand, Traditionalists, the non-educated and those that are native to the area appear tend to place less importance on CREMA outcomes. This appears to be based on a distrust of outside ideas, more direct dependence on resource usage, and a sense that outcomes are divinely ordained. Christianity, education and non-nativity are also associated with much larger performance gaps, particularly with respect to socio-economic impacts.

Respondents in the focus groups described the individual effect of each characteristic with perceptions of CREMA performance somewhat differently. For example, education was seen to raise awareness of the importance of conservation and to open up economic/livelihood alternatives that reduce dependence on natural resources. Christianity, on the other hand, was associated with both a resistance to Traditional belief systems and openness to new ideas. Being non-native to the area was associated with being exposed to—and therefore more open to–new/outside ideas, including CREMAs. In each case, however, the perceived effect was that respondents attached greater importance to CREMA objectives.

These results present an apparent conundrum: the focus group results suggest that those that were not formally educated and/or held Traditional beliefs tend to be the individuals with more reliance on natural resources–yet these characteristics were consistently associated with lower importance scores on the survey. This apparent conundrum can perhaps best be resolved by seeing these patterns as resulting from responses oriented to the CREMA itself, rather than just the specific outcome(s). In other words, the response might be associated with the specific mechanism, and underlying governance regime (CREMA), rather than the outcome itself. Focus groups frequently discussed the ‘openness’ of communities, and their relative exposure to ‘outside’ ideas as explanatory factors for survey results. For many respondents, CREMAs are seen as ‘outside’ forms of conservation/governance that both have the potential to curtail resource use and that contrast with Traditional practices. Some respondents see this outside intervention as a good thing, while others are less sure. Those with more education, for example, may have been exposed to these ideas, and therefore attach more importance to them. For Christians CREMAs may represent an alternative to Traditional beliefs/practices that are not consonant with Christian thought. Traditionalists, on the other hand, may see CREMAs as not consonant with Traditional beliefs and, moreover, ineffective (or irrelevant) in producing outcomes that are precipitated divinely.

A range of scholars have pointed to Traditional religious practices and beliefs as entry points into the design of more effective conservation efforts in Ghana and Africa more generally [13–19, 28]. The results presented here suggest that considerations of the role of religion should extend beyond identifying existing conservation institutions (e.g. taboos) as points of leverage, to considering the way that religio-cultural identity can act as a sort of interpretive lens, through which outside conservation interventions (like CREMAs) are understood and evaluated. At a practical level, this suggests that the shaping of conservation initiatives should involve striking some challenging balances. For example, outside interventions might seek guidance from religious experts and other leaders on ways to incorporate Traditional practices/beliefs in ways that resonate with Traditionalists, but that are palatable to Christians (see for example, [23]). Another challenge lies in taking advantage of the ‘openness’ of some respondents to new ideas without unduly raising expectations (note the much larger performance gaps for some groups) and/or alienating those that may be wary of the utility of those ideas. Notably, there are longer-term trends in Ghana towards the decline of those identifying with Traditional religions; at the same time, many Ghanaians practice ‘hybrid’ forms of religion, where elements of Traditional beliefs/practices are incorporated by those that identify as Christian [18–20, 23]. How will conservation interventions adapt to this changing religious landscape? While challenging, striking these types of balances may also be facilitated by strategically using the flexibility of the CREMA mechanism to incorporate Traditional beliefs and leadership [10]. Whether and how CREMAs are successful in achieving these balances will be important to characterize and monitor in continuing research efforts.

More generally, these results resonate with a growing body of work that suggests that context, including aspects of culture, shapes variations in the perceptions that community stakeholders have with respect to the performance of conservation interventions [10–11, 31–32]. Clearly, perceptions are shaped by factors other than religion or education (or nativity) [10–11] but the findings presented above suggest that the perceptions of groups defined by religion (as well as education and nativity) are *systematically* different. Moreover, these patterns hold at national as well as local levels. These systematic differences in response patterns have implications for using perceptions as a means of monitoring and evaluating PA performance in Ghana and elsewhere [8].

## Supporting information

S1 Table Mean importance and satisfaction responses in the national level survey, with performance gaps for each religious group. (DOCX)

S2 Table Mean importance and satisfaction responses in the national level survey, with performance gaps for those with some school and no school. (DOCX)

S3 Table Mean importance and satisfaction responses in the national level survey, with performance gaps for those that are native to the area and those that are not. (DOCX)

S1 Text Analysis of correlations between religion, education and nativity at the national level. (DOCX)
