# Supplementary material for: Religion and perceptions of community-based conservation in Ghana, West Africa
Source: PLoS One. 2018 Apr 5;13(4):e0195498. doi: 10.1371/journal.pone.0195498 (PMC5886562; doi:10.1371/journal.pone.0195498)
Supplement: S1 Table — (DOCX) [file pone.0195498.s001.docx]

S1 Table: Mean importance /satisfaction scores and performance gaps for Christians and Traditionalists for five sampled CREMAs (n = 476).^a^

| Outcomes^b^ | Importance | | | Satisfaction | | | Gap | |
| --- | --- | --- | --- | --- | --- | --- | --- | --- |
|  | C^c^ | T^d^ | p^e^ | C | T | p | C | T |
| educational scholarships | 4.64 | 3.46 | **<0.001** | 2.08 | 2.8 | **<0.001** | 2.56 | 0.66 |
| access to credit/financial assistance | 4.55 | 3.26 | **<0.001** | 2.15 | 2.87 | **<0.001** | 2.4 | 0.39 |
| improved social infrastructure | 4.59 | 3.51 | **<0.001** | 2.24 | 2.97 | **<0.001** | 2.35 | 0.54 |
| increased income | 4.64 | 3.41 | **<0.001** | 2.42 | 2.8 | 0.065 | 2.22 | 0.61 |
| increased employment | 4.65 | 3.51 | **<0.001** | 2.65 | 2.95 | 0.135 | 2 | 0.56 |
| constancy of kids school attendance | 4.6 | 3.48 | **<0.001** | 2.82 | 2.93 | 0.607 | 1.78 | 0.55 |
| more fish | 4.3 | 3.69 | **<0.001** | 2.63 | 3.41 | **<0.001** | 1.67 | 0.28 |
| capacity building and training in income generating enterprises | 4.57 | 3.7 | **<0.001** | 2.98 | 3.15 | 0.394 | 1.59 | 0.55 |
| international recognition and pride | 4.6 | 4.02 | **<0.001** | 3.01 | 3.82 | **<0.001** | 1.59 | 0.2 |
| improved water supply and quality | 4.69 | 4 | **<0.001** | 3.16 | 3.53 | **0.050** | 1.53 | 0.47 |
| better farmlands, increased food production | 4.61 | 3.92 | **<0.001** | 3.09 | 3.54 | **0.015** | 1.52 | 0.38 |
| more poles and construction materials | 4.42 | 3.69 | **<0.001** | 2.92 | 3.18 | 0.143 | 1.5 | 0.51 |
| more bushmeat | 3.74 | 3.25 | **0.017** | 2.25 | 2.9 | **0.001** | 1.49 | 0.35 |
| tourism | 4.72 | 3.9 | **<0.001** | 3.27 | 3.7 | **0.017** | 1.45 | 0.2 |
| more and better quality traditional medicines | 4.49 | 3.7 | **<0.001** | 3.27 | 3.38 | 0.566 | 1.22 | 0.32 |
| wind break | 4.37 | 3.45 | **<0.001** | 3.24 | 3.26 | 0.891 | 1.13 | 0.19 |
| increased conservation awareness | 4.74 | 4.39 | **0.002** | 3.68 | 4.13 | **0.001** | 1.06 | 0.26 |
| improved supply and quality of firewood and charcoal | 3.64 | 3.44 | 0.228 | 2.58 | 3 | **0.030** | 1.06 | 0.44 |
| native wildlife return | 4.59 | 4.02 | **<0.001** | 3.53 | 3.8 | 0.106 | 1.06 | 0.22 |
| collective community action and unity | 4.55 | 3.66 | **<0.001** | 3.49 | 3.49 | 0.989 | 1.06 | 0.17 |
| more rain | 4.38 | 3.7 | **<0.001** | 3.33 | 3.41 | 0.671 | 1.05 | 0.29 |
| ecologically sensitive areas being protected and well managed | 4.67 | 4.07 | **<0.001** | 3.71 | 3.98 | 0.061 | 0.96 | 0.09 |
| no chemical contamination of water | 4.55 | 3.46 | **<0.001** | 3.63 | 3.28 | 0.069 | 0.92 | 0.18 |
| religious, cultural and historical uses | 4.26 | 3.95 | **0.040** | 3.34 | 3.72 | **0.024** | 0.92 | 0.23 |
| purification and provision of clean air | 4.6 | 3.74 | **<0.001** | 3.71 | 3.43 | 0.103 | 0.89 | 0.31 |
| reduced bush fires | 4.59 | 3.82 | **<0.001** | 3.88 | 3.67 | 0.238 | 0.71 | 0.15 |
| more and better quality grass | 3.95 | 3.92 | 0.840 | 3.3 | 3.62 | 0.059 | 0.65 | 0.3 |
| fodder for livestock | 4.11 | 3.5 | **0.001** | 3.46 | 3.12 | 0.077 | 0.65 | 0.38 |
| Average performance gap (all outcomes) |  |  |  |  |  |  | 1.4 | 0.35 |

^a^ In 419 cases there was no response recorded for this question. While all CREMAs experienced this non-response issue, 342 cases were in Zukpiri (83% item non response) and Wechiau (91% item non-response). Avu Lagoon had 57 non-responses to this question, which is 24.6% of the total response pool. Both Zukpiri and Wechiau are in the Upper West Region where there is likely a higher proportion of Muslims (personal observation). Item non-response was largely due to decisions made by the interviewer to not ask this question, in many cases because of time constraints. In addition, 57 respondents identified as Muslim in the survey. In order to simplify analysis, ratings from those that reported themselves as Muslim were excluded.

^b^ Outcomes arranged by decreasing magnitude of performance gaps for Christians

^c^ Respondents that self-identify as Christian (n= 415)

^d^ Respondents that self-identify as Traditional (n = 61)

^e^ items in bold are significant at the p<.05 level
