# Supplementary material for: Religion and perceptions of community-based conservation in Ghana, West Africa
Source: PLoS One. 2018 Apr 5;13(4):e0195498. doi: 10.1371/journal.pone.0195498 (PMC5886562; doi:10.1371/journal.pone.0195498)
Supplement: S2 Table — (DOCX) [file pone.0195498.s002.docx]

S2 Table: Mean importance /satisfaction scores and performance gaps for respondents with some school (S) and no school (NS) for five sampled CREMAs (n = 924).

| Outcome^a^ | Importance | | | Satisfaction | | | Gaps | |
| --- | --- | --- | --- | --- | --- | --- | --- | --- |
|  | S^b^ | NS^c^ | P^d^ | S | NS | p | S | NS |
| educational scholarships | 4.51 | 4.38 | 0.08 | 2.57 | 3.12 | **<0.001** | 1.94 | 1.26 |
| access to credit/financial assistance | 4.37 | 4.24 | 0.08 | 2.65 | 2.11 | **<0.001** | 1.72 | 2.13 |
| improved social infrastructure | 4.42 | 4.26 | **0.02** | 2.70 | 3.30 | **<0.001** | 1.72 | 0.96 |
| increased income | 4.46 | 4.48 | 0.72 | 2.84 | 3.35 | **<0.001** | 1.62 | 1.13 |
| increased employment | 4.49 | 4.51 | 0.76 | 2.97 | 3.51 | **<0.001** | 1.52 | 1.00 |
| constancy of kids school attendance | 4.40 | 4.41 | 0.83 | 3.18 | 3.45 | **0.01** | 1.22 | 0.96 |
| more poles and construction materials | 4.25 | 3.93 | **<0.001** | 3.10 | 3.39 | **<0.001** | 1.15 | 0.54 |
| capacity building and training in income generating enterprises | 4.34 | 4.29 | **0.48** | 3.22 | 3.55 | **<0.001** | 1.12 | 0.74 |
| improved water supply and quality | 4.45 | 4.31 | **0.04** | 3.38 | 3.52 | 0.15 | 1.07 | 0.79 |
| international recognition and pride | 4.48 | 4.34 | **0.04** | 3.43 | 3.55 | 0.22 | 1.05 | 0.79 |
| more fish | 4.02 | 3.87 | **0.06** | 2.98 | 3.15 | 0.09 | 1.04 | 0.72 |
| better farmlands, increased food production | 4.43 | 4.20 | **<0.001** | 3.41 | 3.38 | 0.78 | 1.02 | 0.82 |
| Tourism | 4.59 | 4.44 | **0.02** | 3.59 | 3.80 | **0.03** | 1.00 | 0.64 |
| more bushmeat | 3.51 | 3.63 | 0.24 | 2.51 | 3.00 | **<0.001** | 1.00 | 0.63 |
| more and better quality traditional medicines | 4.32 | 4.27 | 0.54 | 3.45 | 3.75 | **<0.001** | 0.87 | 0.52 |
| increased conservation awareness | 4.63 | 4.48 | **0.01** | 3.85 | 3.93 | 0.34 | 0.78 | 0.55 |
| improved supply and quality of firewood and charcoal | 3.49 | 3.43 | 0.57 | 2.74 | 3.21 | **<0.001** | 0.75 | 0.22 |
| native wildlife return | 4.45 | 4.22 | **<0.001** | 3.75 | 3.78 | 0.78 | 0.70 | 0.44 |
| collective community action and unity | 4.35 | 4.29 | 0.37 | 3.65 | 3.78 | 0.11 | 0.70 | 0.51 |
| more rain | 4.22 | 4.09 | **0.07** | 3.54 | 3.73 | **0.02** | 0.68 | 0.36 |
| wind break | 4.11 | 4.03 | 0.36 | 3.49 | 3.60 | 0.18 | 0.62 | 0.43 |
| ecologically sensitive areas being protected and well managed | 4.49 | 4.30 | **<0.001** | 3.88 | 3.95 | 0.44 | 0.61 | 0.35 |
| religious, cultural and historical uses | 4.16 | 4.07 | 0.25 | 3.58 | 3.80 | **0.01** | 0.58 | 0.27 |
| fodder for livestock | 3.95 | 3.69 | **<0.001** | 3.39 | 3.62 | **0.02** | 0.56 | 0.07 |
| no chemical contamination of water | 4.33 | 4.23 | 0.18 | 3.81 | 3.88 | 0.39 | 0.52 | 0.35 |
| reduced bush fires | 4.43 | 4.33 | 0.12 | 3.91 | 3.97 | 0.49 | 0.52 | 0.36 |
| purification and provision of clean air | 4.35 | 4.05 | **<0.001** | 3.84 | 3.77 | 0.36 | 0.51 | 0.28 |
| more and better quality grass | 3.93 | 3.95 | 0.76 | 3.47 | 3.98 | **<0.001** | 0.46 | -0.03 |
| Average performance gap (all outcomes) |  |  |  |  |  |  | 0.98 | 0.64 |

^a^ Outcomes arranged by decreasing magnitude of performance gaps for those with some school (S)

^b^ respondents that state they have had some schooling (n= 500)

^c^ respondents that state they have had no schooling (n = 424)

^d^ items in bold are significant at the p<.05 level
