# Supplementary material for: Religion and perceptions of community-based conservation in Ghana, West Africa
Source: PLoS One. 2018 Apr 5;13(4):e0195498. doi: 10.1371/journal.pone.0195498 (PMC5886562; doi:10.1371/journal.pone.0195498)
Supplement: S3 Table — (DOCX) [file pone.0195498.s003.docx]

S3 Table: Mean importance /satisfaction scores and performance gaps for non-natives (NN)^a^ and natives (N) for five sampled CREMAs (n = 699).^b^

| Outcomes^c^ | Importance | | | Satisfaction | | | Gaps | |
| --- | --- | --- | --- | --- | --- | --- | --- | --- |
|  | NN^d^ | N^e^ | P^f^ | NN | N | p | NN | N |
| educational scholarships | 4.75 | 4.28 | **<0.001** | 2.10 | 3.29 | **<0.001** | 2.65 | 0.99 |
| increased income | 4.76 | 4.29 | **<0.001** | 2.40 | 3.41 | **<0.001** | 2.36 | 0.88 |
| increased employment | 4.80 | 4.34 | **<0.001** | 2.47 | 3.53 | **<0.001** | 2.33 | 0.81 |
| access to credit/financial assistance | 4.75 | 4.09 | **<0.001** | 2.04 | 3.35 | **<0.001** | 2.71 | 0.74 |
| improved social infrastructure | 4.66 | 4.15 | **<0.001** | 2.15 | 3.48 | **<0.001** | 2.51 | 0.67 |
| capacity building and training in income generating enterprises | 4.60 | 4.12 | **<0.001** | 2.82 | 3.52 | **<0.001** | 1.78 | 0.60 |
| constancy of kids school attendance | 4.78 | 4.02 | **<0.001** | 2.72 | 3.48 | **<0.001** | 2.06 | 0.54 |
| improved water supply and quality | 4.68 | 4.15 | **<0.001** | 2.95 | 3.66 | **<0.001** | 1.73 | 0.49 |
| international recognition and pride | 4.58 | 4.31 | **0.01** | 2.47 | 3.85 | **<0.001** | 2.11 | 0.46 |
| better farmlands, increased food production | 4.66 | 4.15 | **<0.001** | 2.85 | 3.70 | **<0.001** | 1.81 | 0.45 |
| more and better quality traditional medicines | 4.40 | 4.10 | **<0.001** | 3.29 | 3.73 | **<0.001** | 1.11 | 0.37 |
| more poles and construction materials | 4.35 | 3.85 | **<0.001** | 2.60 | 3.49 | **<0.001** | 1.75 | 0.36 |
| increased conservation awareness | 4.77 | 4.36 | **<0.001** | 3.41 | 4.03 | **<0.001** | 1.36 | 0.33 |
| more fish | 4.38 | 3.66 | **<0.001** | 2.34 | 3.33 | **<0.001** | 2.04 | 0.33 |
| tourism | 4.65 | 4.38 | **<0.001** | 3.15 | 4.05 | **<0.001** | 1.50 | 0.33 |
| collective community action and unity | 4.72 | 4.09 | **<0.001** | 3.35 | 3.84 | **<0.001** | 1.37 | 0.25 |
| more bushmeat | 4.06 | 3.17 | **<0.001** | 2.35 | 2.95 | **<0.001** | 1.71 | 0.22 |
| fodder for livestock | 3.82 | 3.69 | 0.52 | 3.47 | 3.47 | 0.99 | 0.35 | 0.22 |
| reduced bush fires | 4.61 | 4.21 | **<0.001** | 3.64 | 3.99 | 0.03 | 0.97 | 0.22 |
| native wildlife return | 4.67 | 4.11 | **<0.001** | 3.30 | 3.91 | **<0.001** | 1.37 | 0.20 |
| more and better quality grass | 3.94 | 3.96 | 0.91 | 3.69 | 3.82 | 0.57 | 0.25 | 0.14 |
| improved supply and quality of firewood and charcoal | 3.56 | 3.28 | 0.06 | 2.65 | 3.15 | **<0.001** | 0.91 | 0.13 |
| more rain | 4.42 | 3.94 | **<0.001** | 3.42 | 3.81 | **0.01** | 1.00 | 0.13 |
| wind break | 4.41 | 3.81 | **<0.001** | 3.31 | 3.68 | **0.01** | 1.10 | 0.13 |
| ecologically sensitive areas being protected and well managed | 4.68 | 4.21 | **<0.001** | 3.45 | 4.08 | **<0.001** | 1.23 | 0.13 |
| no chemical contamination of water | 4.55 | 4.04 | **<0.001** | 3.72 | 3.92 | 0.23 | 0.83 | 0.12 |
| religious, cultural and historical uses | 4.32 | 4.00 | **<0.001** | 3.40 | 3.88 | **<0.001** | 0.92 | 0.12 |
| purification and provision of clean air | 4.52 | 3.94 | **<0.001** | 3.47 | 3.85 | **<0.001** | 1.05 | 0.09 |
| Average performance gap (all outcomes) |  |  |  |  |  |  | 1.53 | 0.37 |

^a^ Respondents were asked if they were originally from the area – those that were categorized as Native, those that weren’t as non-native.

^b^ In 230 cases there was no response recorded for this question. This was due to the length of the survey and item non-response did not systematically skew the data.

^c^ Outcomes arranged by decreasing magnitude of performance gaps for non-natives

^d^ respondents that identify as non-native to the area (n = 93)

^e^ respondents that identified as native to the area (n = 606)

^f^ items in bold are significant at the p<.05 level
