# Supplementary material for: Religion and perceptions of community-based conservation in Ghana, West Africa
Source: PLoS One. 2018 Apr 5;13(4):e0195498. doi: 10.1371/journal.pone.0195498 (PMC5886562; doi:10.1371/journal.pone.0195498)
Supplement: S1 Text — (DOCX) [file pone.0195498.s004.docx]

S4 Text: Tests of correlation between religion, education, and ‘nativity’ at the national level (5 CREMAs)

In order to check for relationships between these religion, education and nativity, chi square tests were run. Tests involving religion did not include Muslims. Religion and Nativity (n=282) were found to be correlated; those that were not native to the areas were not likely to be Traditionalist (Pearson chi-square = 10.182; df 1; p=.001). This was a weak effect (phi = -.190). Religion and education (n= 476) were also correlated, with more Christians tending to have had some schooling (Pearson chi-square = 35.002; df=1; p <.001). The effect size for this relationship was moderate (phi = -.272). Education and nativity (n=279) were also found to be correlated; those with no schooling tended to be native to the area (Pearson chi square = 6.932; df=1, p=.008). This was a relatively weak effect (phi = -.158).
